# Common occurrence of hotspots of single strand DNA breaks at transcriptional start sites

**DOI:** 10.1186/s12864-024-10284-4

**Published:** 2024-04-15

**Authors:** Huifen Cao, Yufei Zhang, Tianrong Song, Lu Xia, Ye Cai, Philipp Kapranov

**Affiliations:** 1 Institute of Genomics, School of Medicine, Huaqiao University, 668 Jimei Road, 361021 Xiamen, China; 2 Xiamen Cell Therapy Research Center, The First Affiliated Hospital of Xiamen University, 361000 Xiamen, China; 3 State Key Laboratory of Cellular Stress Biology, School of Life Sciences, Xiamen University, 361102 Xiamen, China

**Keywords:** Breakome, Single strand DNA break, Abasic site, Transcription start site, Hotspot

## Abstract

**Background:**

We recently developed two high-resolution methods for genome-wide mapping of two prominent types of DNA damage, single-strand DNA breaks (SSBs) and abasic (AP) sites and found highly complex and non-random patterns of these lesions in mammalian genomes. One salient feature of SSB and AP sites was the existence of single-nucleotide hotspots for both lesions.

**Results:**

In this work, we show that SSB hotspots are enriched in the immediate vicinity of transcriptional start sites (TSSs) in multiple normal mammalian tissues, however the magnitude of enrichment varies significantly with tissue type and appears to be limited to a subset of genes. SSB hotspots around TSSs are enriched on the template strand and associate with higher expression of the corresponding genes. Interestingly, SSB hotspots appear to be at least in part generated by the base-excision repair (BER) pathway from the AP sites.

**Conclusions:**

Our results highlight complex relationship between DNA damage and regulation of gene expression and suggest an exciting possibility that SSBs at TSSs might function as sensors of DNA damage to activate genes important for DNA damage response.

**Supplementary Information:**

The online version contains supplementary material available at 10.1186/s12864-024-10284-4.

## Background

DNA plays a crucial role of being the central information repository inside a living cell, yet its chemical and physical integrity is constantly challenged by exogenous and endogenous factors that can result in multiple types of DNA damage [1]. Unrepaired or improperly repaired DNA damage can lead to serious consequences to the cell and whole ogranism, as shown by the vast body of evidence that connects DNA damage to aging, cancer, neurological and many other diseases [2–4]. The cornerstone importance of DNA damage for basic biology and human disease has created significant amount of research interest into analysis of various types of DNA damage at the genome level [5–8] in order to understand the intricate details of how different lesions impact cell physiology. Recently, our group has published the development and application of SSiNGLe and SSiNGLe-AP methods for high-resolution genome-wide profiling of two most abundant types of DNA damage, single-strand DNA breaks (SSBs) and abasic (AP) sites respectively [9, 10]. One of the major outcomes from these studies was the realization that the distribution of these two lesions in mammalian genomes is not random [9, 10]. One of the manifestation of this phenomenon is the existence of single-nucleotide hotspots of SSBs and AP sites [9, 10]. Moreover, we have shown that the hotspots of SSBs have a tendency to occur in the vicinity of TSSs of genes based on analysis of SSB profiles in two human cell types [11].

DNA damage has a complex and intertwined relationship with gene expression. While the progression of RNA polymerases can be blocked by various types of DNA lesions, which can be caused in part by the act of transcription itself, this process also requires programmed DNA damage for proper regulation and execution [12]. In this respect, the association of DNA breaks with TSSs is consistent with an emerging theme of DNA damage as a regulator of gene expession based on a number of studies that have shown that DNA breaks [13–22] and AP sites [23, 24] formed at promoters or enhancers can regulate transcription. For example, the study by Ju et al. [13] have shown that a site-specific double-strand DNA break (DSB) induced in the *pS2* promoter by topoisomerase IIβ (TOP2β) is required for transcriptional activation. In another example, induction of DNA breaks in the immediate vicinity of the TSS of the p21 gene by caspase-activated DNase (CAD) during a cellular differentiation program was shown to be critical for the transcriptional activation of p21 [19]. In addition to DNA breaks, AP sites found in promoters in the context of G-rich sequences capable of forming G-quadruplexes have been shown to regulate gene expression via attracting prolonged binding of multifunctional apurinic/apyrimidinic endonuclease 1 (APE1) protein that can function as a transcriptional trans activator [25].

However, a significant amount of controversy exists in regards to the relatinship between DNA damage, specifically DNA breaks, located at promoters and TSSs and transcription activation. First, DNA breaks, either DSBs or SSBs, formed at promoters are well-documented to inhibit transcription [26–31], reviewed in [32]. Second, while the enrichment of DSBs at TSSs was found by several groups and different methods, the enrichment of SSBs around TSSs is more controversial. As mentioned above, we recently reported the enrichment of SSBs, and especially SSB hotspots, in the ± 200 bp regions around TSSs using SSiNGLe, albeit in just two cell types one of which was a cancer cell line [11]. Interestingly, the presence of TSS-SSBs (SSBs or their hotspots found near TSSs) positively correlated with gene expression [11]. These results were consistent with those from a previous study that used a lower resolution method SSB-Seq to map SSBs genome-wide [33]. On the other hand, a different study that used another high-resolution method GLOE-seq obtained opposite conclusions — depletion, rather than enrichment, of SSBs around human TSSs [34]. Therefore, in this study, we have significantly expanded on our original observations concerning the existence of SSB hotspots and their enrichment around TSSs by analyzing genome-wide profiles of SSBs, AP sites and transcriptomes from 6 normal mouse tissues recently published by our group [10].

## Results

### SSB hotspots exist in normal mammalian tissues

**Fig. 1:**
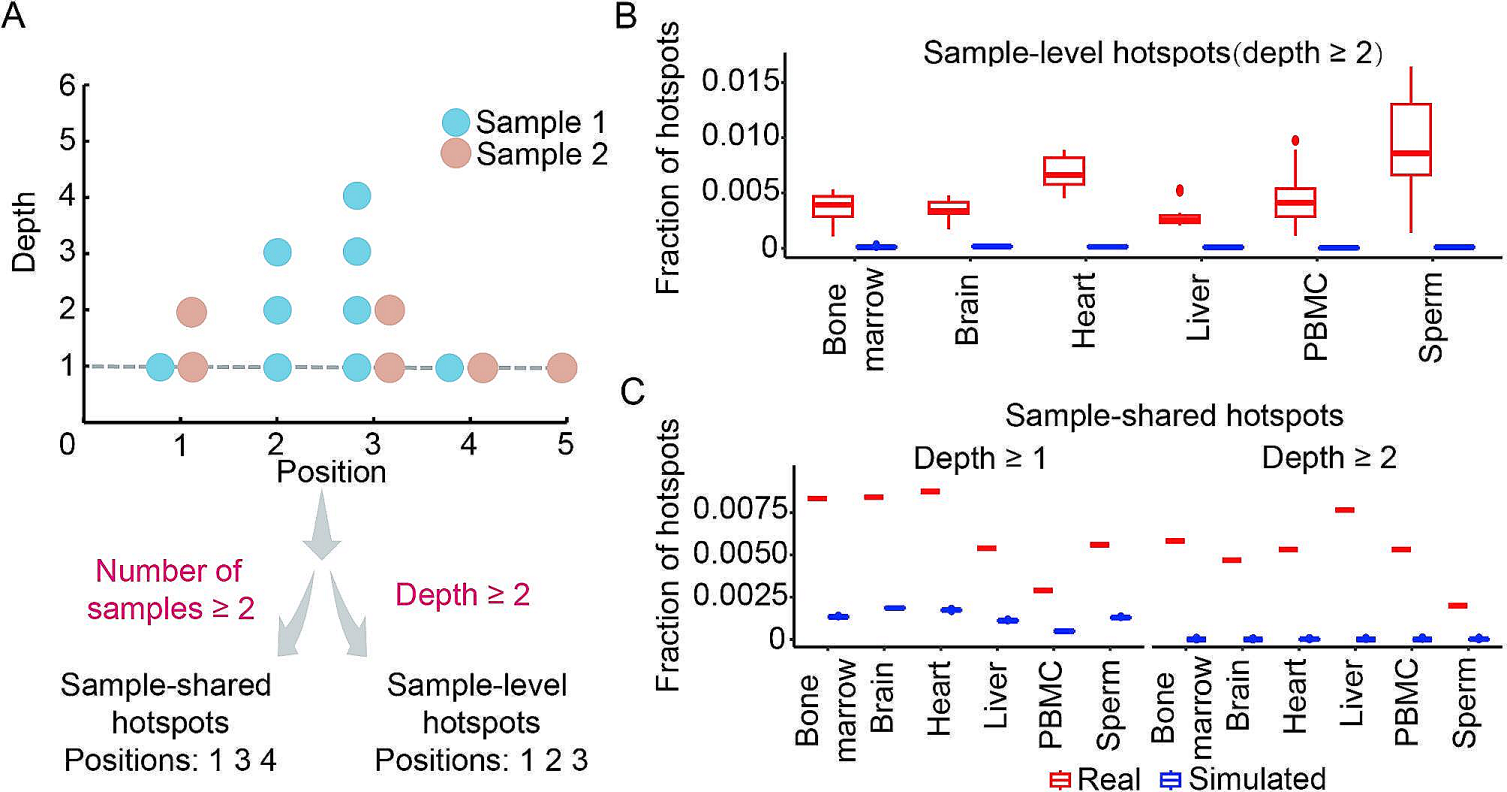
SSB hotspots exist in all tested mouse tissues. (**A**) The diagram showing the definition of the sample-level and sample-shared hotspots. (**B**, **C**) The fractions of sample-level (**B**) and sample-shared (**C**) hotspots in mouse genome (Y-axes) for the real (red) and simulated (blue) data in each tissue (X-axes). (**B**, **C**) Box plots indicate median (middle line), 25th, 75th percentile (box) and 1.5× interquartile range (whiskers) as well as outliers (single points)

We previously reported that nucleotide-level hotspots of DNA damage can be defined in at least two ways as illustrated in the Fig. 1A: (1) in the same sample based on detection by multiple reads (sample-level hotspots) and (2) in different samples but with one or more reads (sample-shared hotspots) [10]. We assumed that detection of a DNA break at the same nucleotide position with one read, but in 2 independent biological samples would be a more stringent hotspot criterion compared to detection with 2 reads, but in the same sample. Thus, defining hotspots in different ways allows for varying the stringency of the resulting hotspots which in turn can allow for more reliable detection of the true genomic patterns that are characteristic of the hotspots. On the other hand, making the stringency too high would result in too few hotspots available for a reliable analysis. Therefore, in this work, we first have defined SSB hotspots using 3 different criteria in the non-repeat portion of the mouse genome (Methods).

First, sample-level hotspots were defined as nucleotide positions where SSBs could be found by at least 2 reads (depth ≥ 2) in each of the 71 samples representing 6 normal mouse tissues or cell types — brain, liver, heart, sperm, bone marrow and PBMC — genome-wide SSB profiles of which have been published by us recently [10]. Each tissue type was represented by 12 samples (with the exception of brain that had 11) — 3 different animals for each of the 4 age groups (3, 12, 19 and 22 months) [10]. Importantly, PCR duplicates were removed in each sample prior to the hotspot detection to ensure that the hotspots were represented by independent DNA molecules. Starting with respectively 8,888,701, 6,414,353, 8,357,645, 5,367,568, 2,341,837 and 6,199,132 SSBs in brain, bone marrow, heart, liver, PBMC and sperm, respectively, we could identify correspondingly 31,537, 27,112, 56,605, 15,925, 12,213 and 61,067 sample-level hotspots (Supplementary Table 1). Second, we could identify 6,800 − 74,853 sample-shared hotspots detected by at least one read (depth ≥ 1) in at least two independent samples of each tissue. Third, by further increasing the stringency to requirement for the sample-shared hotspots to be detected by at least 2 reads (depth ≥ 2) in at least two independent samples of each tissue, as expected, we found much fewer, 65–301, positions represented only 0.2–0.96% of the sample-shared hotspots found with the depth ≥ 1 (Supplementary Table 1).

However, SSBs can be found multiple times at the same genomic positions in the same or different samples purely by random chance. Therefore, to investigate whether the observed hotspots were real, we then estimated the fraction of SSBs coordinates of which would be expected to overlap by random chance given the number of SSBs found in our samples. We performed 100 rounds of simulations where in each round hotspots were generated using the same procedure for the same number samples containing the same number of breaks genomic coordinates of which were generated randomly (Methods). As shown in Fig. 1B and C, the fractions of sample-level or sample-shared hotspots for both depths of ≥ 1 or ≥ 2 were always much higher in the real than in the simulated data (p-value < 2.16E-16, two-sided Student t-test, Supplementary Table 2). In the case of the sample-shared hotspots, the differences of real vs. the simulated data increased with the increase in stringency (Fig. 1C, Supplementary Table 2). For example, the ratios of fractions of sample-shared depth ≥ 1 hotspots on the real vs. simulated data in different tissues were in the range of 4.36–6.23 (Fig. 1C, Supplementary Table 2). The corresponding ratios increased significantly to 135.55–1629.22 with read depth ≥ 2 (Fig. 1C, Supplementary Table 2). In summary, these results strongly argued that nucleotide-level hotspots of SSBs do exist in all 6 tested tissues.

### SSB hotspots are enriched in the immediate vicinity of TSSs with preference for the template strand

**Fig. 2:**
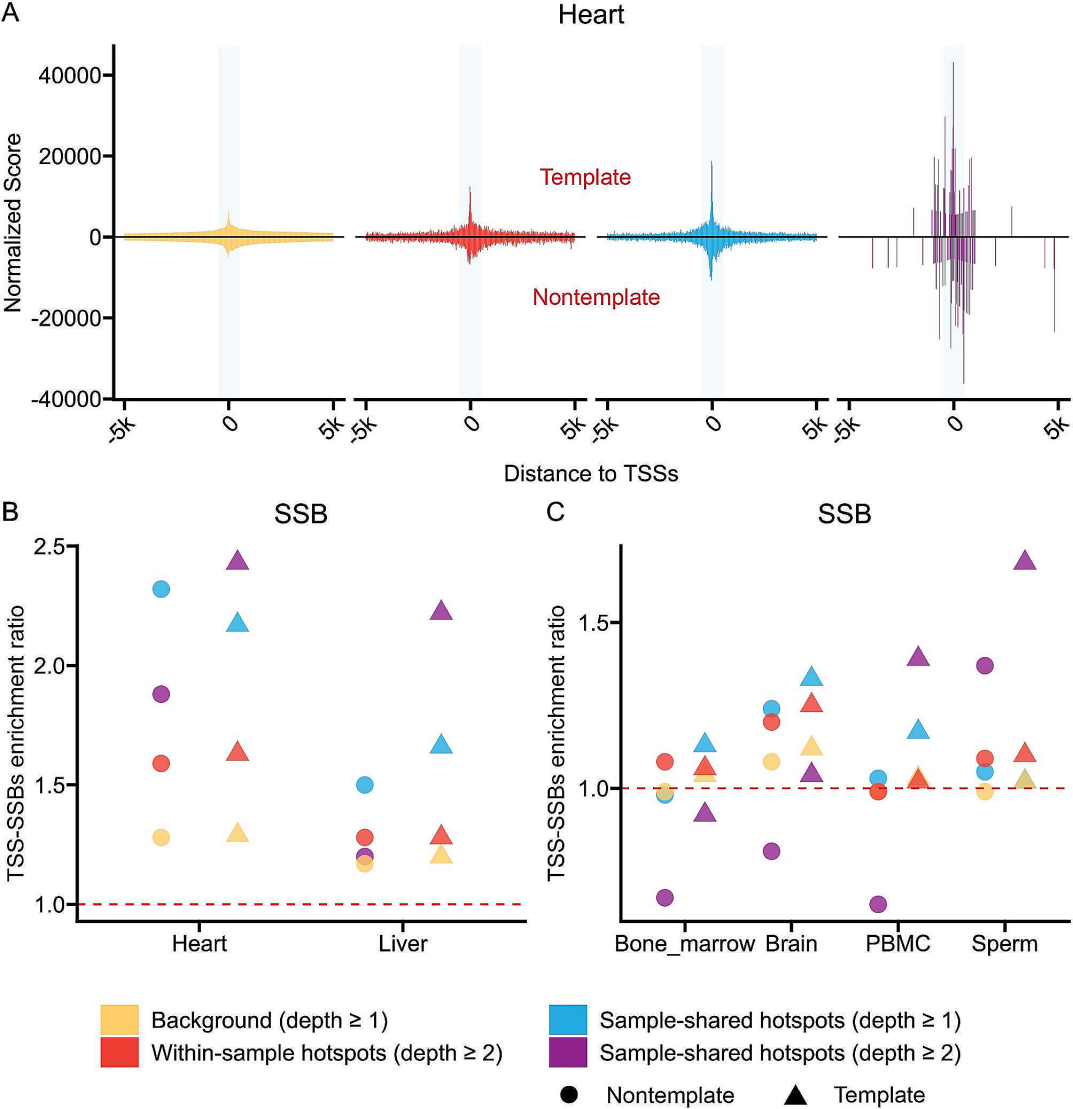
SSB hotspots are significantly enriched around TSSs in different tissue types. (**A**) Aggregate plots of the normalized density scores (Y-axes) of the positions of all SSBs and various SSB hotspots within ± 5,000 bp of TSSs in heart. The opaque vertical rectangles represent the ± 200 bp regions around the TSSs. The signal from the template and nontemplate strands is shown respectively above and below the zero lines. (**B**, **C**) The TSS-SSBs enrichment ratios (Y-axes) for all SSBs (background) and SSB hotspots on either template or non-template strands for different tissues: heart and liver (**B**) and the other four tissues (**C**). Yellow represents background (all SSBs), red represents sample-level hotspots (depth ≥ 2), blue represents sample-shared hotspots (depth ≥ 1) and purple represents sample-shared hotspots (depth ≥ 2). Note, two data points in sperm for the sample-shared hotspots (depth ≥ 1) and background have the same values (Supplementary Table 3), thus only the latter is shown

To investigate the enrichment of SSBs and SSB hotspots in the vicinity of TSSs, similar to our previous study [11], we initially calculated the cumulative densities of all SSBs or SSB hotspots in 20 bp bins around ± 5,000 bp of all annotated TSSs of all mouse genes for each tissue and normalized them to the total number of breaks or hotspots in that tissue (Methods). This analysis was done separately for the template and nontemplate strands. Using this analysis, we observed obvious enrichment of SSBs and all types of SSB hotspots around TSSs on both template and nontemplate strands in heart, as shown in Fig. 2A. To quantify this enrichment and to test whether it exists in other mouse tissues, we then calculated the TSS-SSBs enrichment ratio [11] defined as the number of SSBs mapping to ± 200 bp of TSSs relative to the number of SSBs found around ± 5,000 bp of TSSs for each tissue (Methods).

As illustrated in the Fig. 2B and C (see Supplementary Table 3 for the details), we indeed observed a general enrichment of SSBs and their hotspots around TSSs in all tissues with the following interesting properties. First, as expected, there was a general trend of higher enrichment of hotspots compared to the background (all SSBs detected by at least 1 read) and increasing enrichment with increasing stringency of the SSB hotspots. The sample-level hotspots were more significantly more enriched than the background breaks on either template or nontemplate strands as illustrated by the corresponding median TSS-SSBs enrichment ratios of 1.15 vs. 1.06 (p-value 2.53E-3, paired single-sided Wilcoxon signed rank test, Fig. 2B and C, Supplementary Table 3). On the other hand, the sample-shared hotspots with depth ≥ 1 were even more enriched with the median ratios of 1.20 which was significantly higher (p-value 2.96E-2, paired single-sided Wilcoxon signed rank test) compared with the sample-level hotspots (Fig. 2B and C, Supplementary Table 3). Moreover, in 4 out of 6 tissues, the highest enrichment was observed for the sample-shared hotspots found with the depth of ≥ 2 and on the template strand with the ratios being in the range of 1.39–2.43, median 1.95 (Fig. 2B and C, Supplementary Table 3).

Second, we observed tissue-specific differences in the magnitude of the enrichment, with heart and liver having the highest TSS-SSBs enrichment ratios either for singleton SSBs or hotspots (Fig. 2B, Supplementary Table 3). Finally, we found a significant tendency for all types of SSB hotspots to be higher enriched on the template strands across all 6 tissues compared to the nontemplate strands (p-value 1.94E-3, paired single-sided Wilcoxon signed rank test, Fig. 2B and C, Supplementary Table 3).

Overall, these results have shown a consistent tendency of SSB hotspots to associate with the ± 200 bp regions around TSSs thus supporting and expanding our previous findings [11]. However, in the current study, we further explored enrichment of SSB hotspots in the immediate vicinity of TSSs by splitting the ± 200 bp regions into finer distance bins of ± 5 bp, ± 6–20 bp, ± 21–50 bp, ± 51–100 bp, ± 101–150 bp and ± 151–200 bp. To simplify the interpretation of the data, we limited this, and all other analyses presented below, to only one type of hotspots — sample-shared hotspots found with read depth ≥ 1 for the following two reasons. First, as mentioned above, sample-shared hotspots were, by definition, more stringent than sample-level hotspots which was also observed in the higher enrichment ratios. Second, even though sample-shared hotspots found with the depth ≥ 2 have shown the highest enrichments near TSSs at some tissues, their numbers were too small for the downstream analyses.

**Fig. 3:**
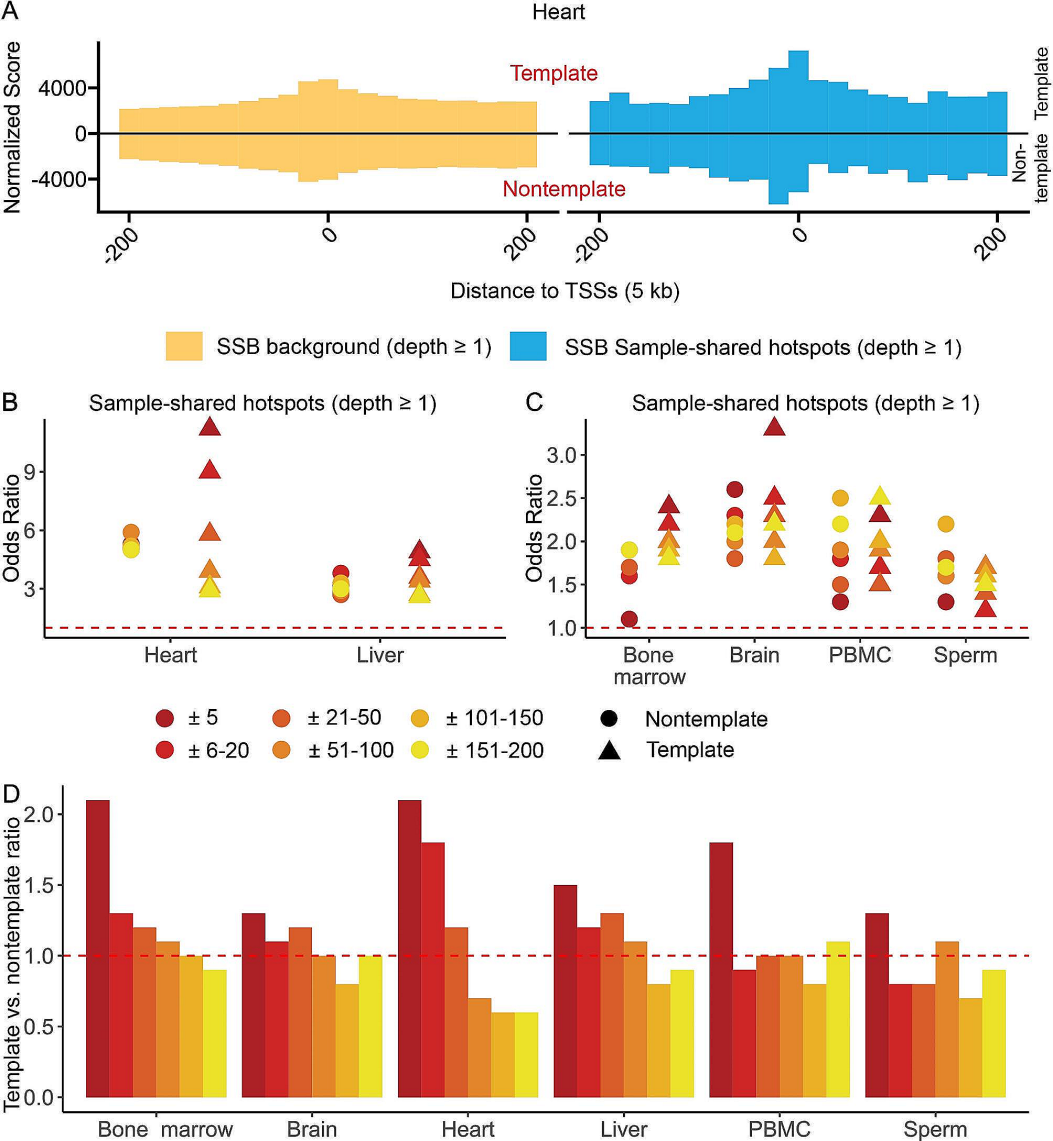
SSB hotspots are enriched on the template strand in the immediate vicinity of TSSs. (**A**) Aggregate plots of the normalized density scores (Y-axes) of the positions of all SSBs or sample-shared hotspots (depth ≥ 1) within ± 200 bp of the TSSs for heart (**A**). The signal from the template and nontemplate strands is shown respectively above and below the zero lines. (**B**, **C**) Odds ratios (Y-axes) of enrichment of sample-shared SSB hotspots (depth ≥ 1) found on other template or non-template strands in the indicated non-overlapping distance bins to TSSs in the different tissues (X-axes). Due to the differences in the magnitudes of the odds ratios, the Y-axes in (**B**) and (**C**) have different scales. (**D**) The template vs. nontemplate ratios (Y-axes) of SSB hotspots in each distance and each tissue. (**B**–**D**) The red dashed horizontal lines represent odds ratios (**B**, **C**) or template vs. nontemplate ratio of 1 that represent respectably no enrichment or no strand preference. See Supplementary Table 3 for the exact numbers and Methods for more details

Interestingly, we observed a strong enrichment of SSB hotspots in the immediate vicinity of TSSs on the cumulative density plots shown in Fig. 3A for the heart. We then calculated the odds ratios of enrichment of the SSB hotspots in each distance bin in each tissue. Strikingly, as can be seen in the Fig. 3B and C (details in Supplementary Table 4), we observed progressive increase in the odds ratios with the decreasing distance around TSS with the highest odds ratios found in the ± 5 bp bins in 4 out of 6 tissues. Furthermore, we found a tendency of SSB hotspots to preferentially occur on the template strands of genes with the decreasing distance to the TSSs in all 6 tissues. For example, the template vs. nontemplate ratios in the ± 5 bp bin ranged from 1.28 to 2.15 (median 1.65) and were significantly larger than those in the ± 151–200 bp bin that ranged from 0.58 to 1.11 (median 0.92) with the p-value 1.56E-2 (paired single-sided Wilcoxon signed rank test, Fig. 3B and C, Supplementary Table 4). Furthermore, we again observed differences among the tissues with heart having the highest odds ratio of enrichment of SSB hotspots on the template strand within the ± 5 bp window around the TSSs followed by liver (Fig. 3B and C, Supplementary Table 4). Thus, SSB hotspots were not only enriched near TSSs — they were also enriched in the immediate vicinity (± 5 bp) around TSSs and on the template strands of the corresponding genes (Fig. 3D, Supplementary Table 4). However, we could not find the SSB hotspot enrichment at a specific position within the ± 5 bp window (Supplementary Table 5), suggesting that while enriched very close to TSSs, SSB hotspots do not favor any specific position in the immediate vicinity of a TSS.

### Presence of proximal template strand TSS-SSBs hotspots associates with higher levels of gene expression

**Fig. 4:**
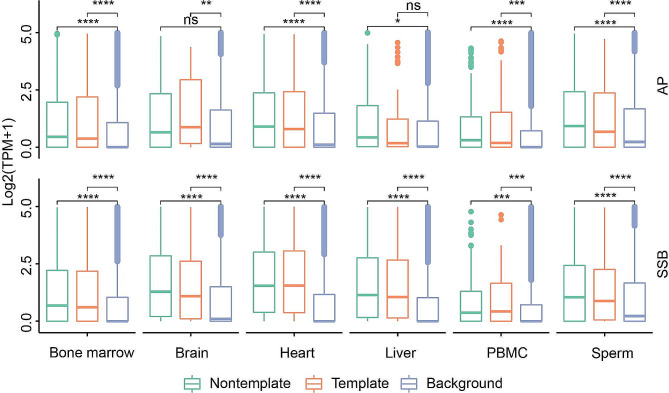
Sample-shared SSB and AP site hotspots found within ± 200 bp of TSSs tend to associate with highly expressed genes. The box plots of the expression levels (log_2_(TPM + 1), Y-axes) of genes with and without sample-shared (depth ≥ 1) AP sites (top) or SSB hotspots (bottom) within ± 200 bp of their TSSs on either template or nontemplate strands are shown for each tissue (X-axes) for AP sites and SSBs. Box plots indicate median (middle line), 25th, 75th percentile (box) and 1.5× interquartile range (whiskers) as well as outliers (single points). The asterisks above the horizontal lines denote the significance of the difference: *, **, ***, **** represent respectively p-values < 0.05, 0.01, 0.001, 0.0001 (Wilcoxon Rank Sum Test), while “ns” mean no significant difference. See Supplemental Tables 6 and Methods for more details

We previously reported based on the two human cell types [11] that genes with SSB hotspots found within ± 200 bp around the TSSs were expressed higher than those without the SSB hotspots. In this work, we further investigated the correlation between the presence of SSB hotspots and genes expression by taking advantage of the RNA-seq data obtained from the same exact 71 samples as the corresponding SSB maps from the 6 normal mouse tissues [10]. For each of the 55,228 mouse genes, we calculated the corresponding expression values in each of the 6 tissues. We then first compared the expression levels of genes with and without SSB hotspots on either strand in the ± 200 bp windows around the TSSs and found that in each tissue, genes with the hotspots had significantly higher expression levels compared to genes without the hotspots (Fig. 4, Supplementary Table 6). However, we found no differences in the expression levels of genes that contained SSB hotspots on the template strands in the ± 200 bp windows around the TSSs vs. those that contained SSB hotspots on the nontemplate strand (Fig. 4, Supplementary Table 6).

**Fig. 5:**
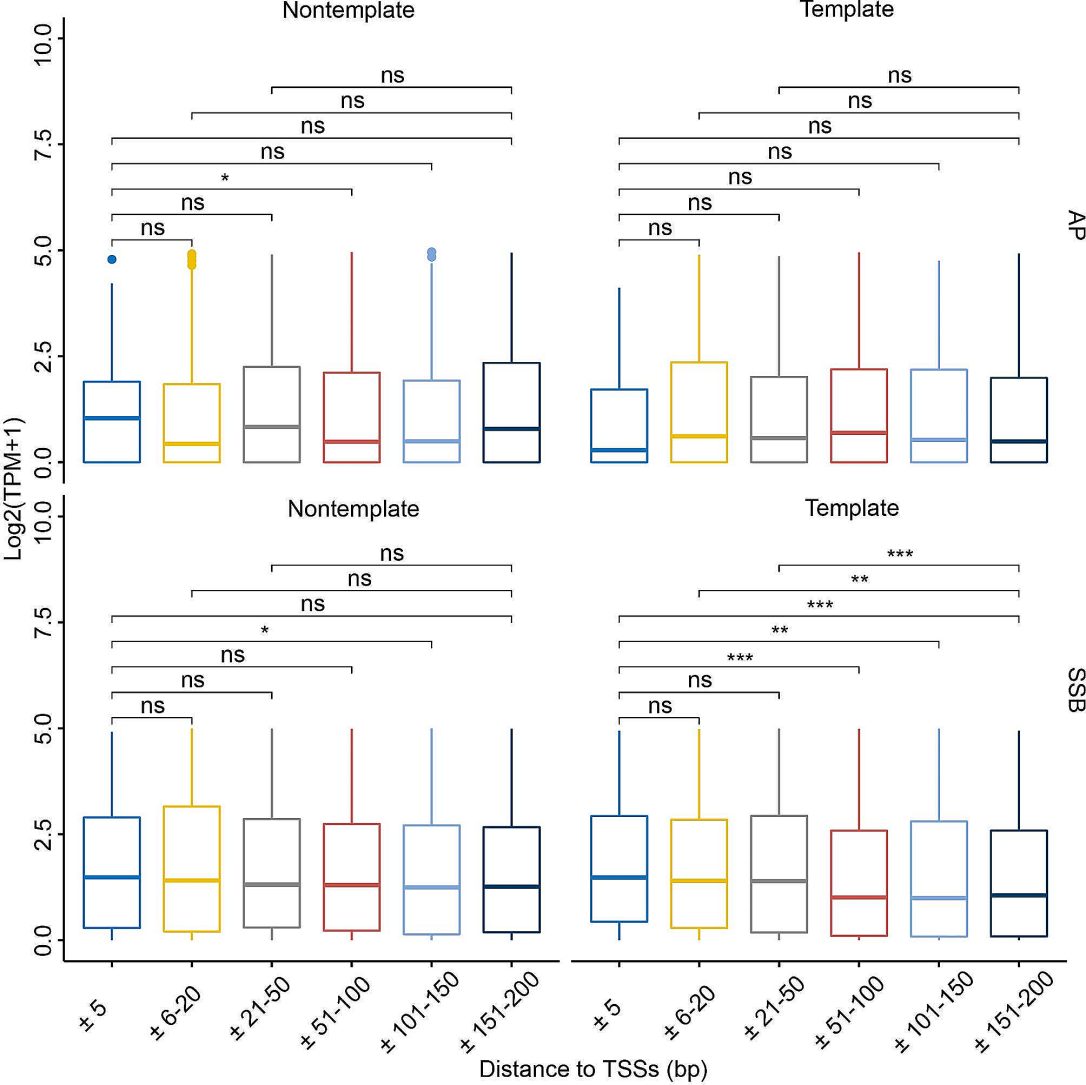
Closer proximity of template strand SSB hotspots is associated with higher gene expression. Box plots of the expression levels (log2(TPM + 1), Y-axes) of genes that contain sample-shared (depth ≥ 1) hotspots of AP sites (A) and SSBs (B) within the indicated non-overlapping bins (X-axes) near their TSSs on the template or non-template strands in at least one tissue. Box plots indicate median (middle line), 25th, 75th percentile (box) and 1.5× interquartile range (whiskers) as well as outliers (single points). The asterisks above the horizontal lines denote the significance of the difference: *, **, *** represent respectively p-values < 0.05, 0.01 and 0.001 (Wilcoxon Rank Sum Test), while “ns” mean no significant difference. See Supplemental Tables 6 and Methods for more details

Second, we explored whether decreasing the distance between SSB hotspots and TSSs had any effect on gene expression by comparing the expression levels of genes that contained SSB hotspots on either the template or nontemplate strand in the ± 5 bp, ± 6–20 bp, ± 21–50 bp, ± 51–100 bp, ± 101–150 bp and ± 151–200 bp bins around the corresponding TSS. For this analysis, we combined all expression data from all tissues in order to have sufficient power for the analysis (Methods). Strikingly, as shown in the Fig. 5, we have observed progressive increase in the expression of genes with the decreasing distance between the hotspots located on the template strand and the TSSs. Genes containing hotspots on the template strand and within the ± 5 bp windows around the TSSs had significantly higher expression than the genes with the hotspots in the 3 distance bins: ± 51–100 bp, ± 101–150 bp and ± 151–200 bp (Fig. 5, Supplementary Table 6). Furthermore, while not significant, the expression of genes with hotspots in the ± 5 bp window was higher than that of genes with hotspots in the ± 6–20 bp and ± 21–50 bp windows (Fig. 5, Supplementary Table 6). Interestingly, we also observed similar trend for genes containing SSB hotspots on the nontemplate strands albeit the differences were not statistically significant (Fig. 5, Supplementary Table 6). Thus, even though we found no differences in the expression levels between the strands in the ± 200 bp windows around TSSs, we could find them in the more narrow windows.

### SSB hotspots associate with a specific subset of TSSs

As the next step, we investigated whether the presence of SSB hotspots within ± 5 bp windows around TSSs is an innate feature of all or just a subset of mouse genes. Overall, we could identify 1,106 TSSs which contained such hotspots, corresponding to 854 or 1.55% of all annotated 55,228 mouse genes. However, the number of TSSs and genes associated with SSB hotspots would likely increase with increase in the depth of sequencing and inclusion of additional tissue types, and therefore can not be used to estimate the actual number of genes with the SSB hotspots at their TSSs. Therefore, to answer this question, we used the 854 genes to investigate whether genes with the TSS-associated SSB hotspots had certain features that differentiated them from the rest of genes.

**Fig. 6:**
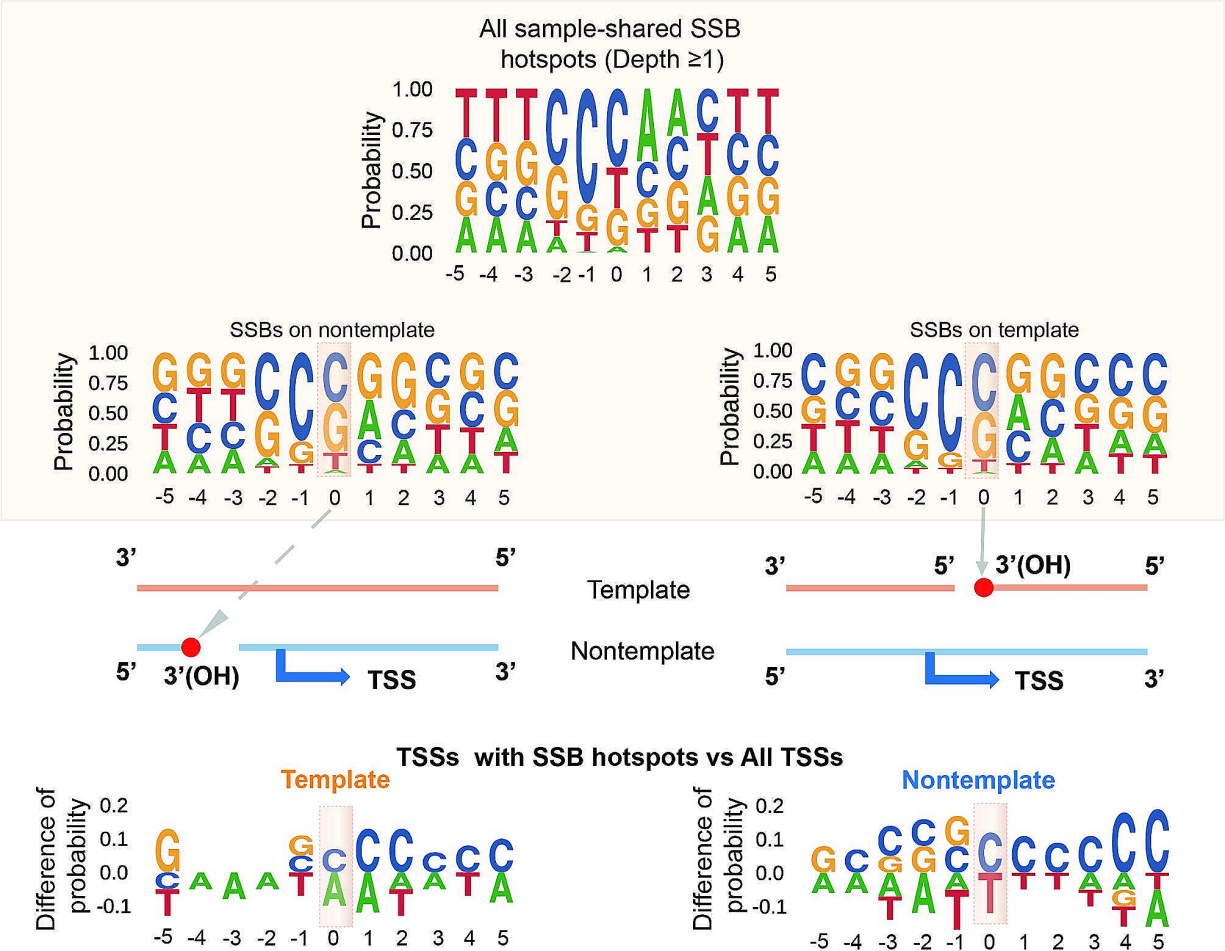
SSB hotspots have a tendency to be found downstream of cytosines leading to cytosine enrichment of regions around TSSs associated with the hotspots. The top 3 sequence motifs represent motifs around all SSB hotspots found genome-wide and only those found on either template or non-template strands in ± 5 bp vicinity around TSSs. The bases immediately upstream of the breaks are highlighted. The bottom two motifs represent the difference in the sequence compositions around TSSs that contain SSB hotspots on either template or non-template strand vs. the sequences around all TSSs. Only bases with differences > 0.05 (5%, Y-axes) at each position are shown. Positive values on Y-axes indicate enrichment in the TSSs with the SSB hotspots. The TSSs are highlighted. See Supplemental Tables 7 and Methods for more details

We previously found that human SSBs tend to occur immediately downstream of a short cytosine-rich motif [11]. As shown in the Fig. 6, we found similar sequence profiles for the mouse sample-shared SSB hotspots found either anywhere in the genome or near TSSs. Furthermore, the sequences in the vicinity of TSSs associated with such SSB hotspots were also enriched in cytosines on the strand that contained the hotspots (Fig. 6, Supplementary Table 7). The median cytosine fraction on the template strands in the ± 5 bp windows around TSSs of all genes was 28%, however, it increased to 40% for TSSs that contained SSB hotspots on the template strands and this increase was significant (p-value 4.88E-4, paired single-sided Wilcoxon signed rank test, Supplementary Table 7). Likewise, the median fraction of cytosines increased on the nontemplate strands around TSSs associated with SSB hotspots on these strands relative to the corresponding value for all genes: 32% vs. 27% (p-value 6.83E-3, paired single-sided Wilcoxon signed rank test, Supplementary Table 7).

The differences in the sequence motifs suggested that while the presence of TSS-proximal SSB hotspots on the template strand is a feature of many genes, it may not be a universal attribute of all genes. As the next step, we explored whether the genes with TSS-proximal SSB hotspots were enriched in specific functions. We performed Gene Ontology (GO) analysis with the 854 genes using all annotated mouse genes as the background and found striking enrichment of multiple terms associated with RNA processing and, interestingly, DNA damage response, such terms as “ncRNA processing”, “mRNA processing” and “regulation of response to DNA damage stimulus” (Supplementary Table 8). Thus, SSB hotspots appear to have a tendency to associate with TSSs of a specific subset of genes rather than with TSSs of all genes.

### TSS-associated features of SSB hotspots are different from those of AP site hotspots

**Fig. 7:**
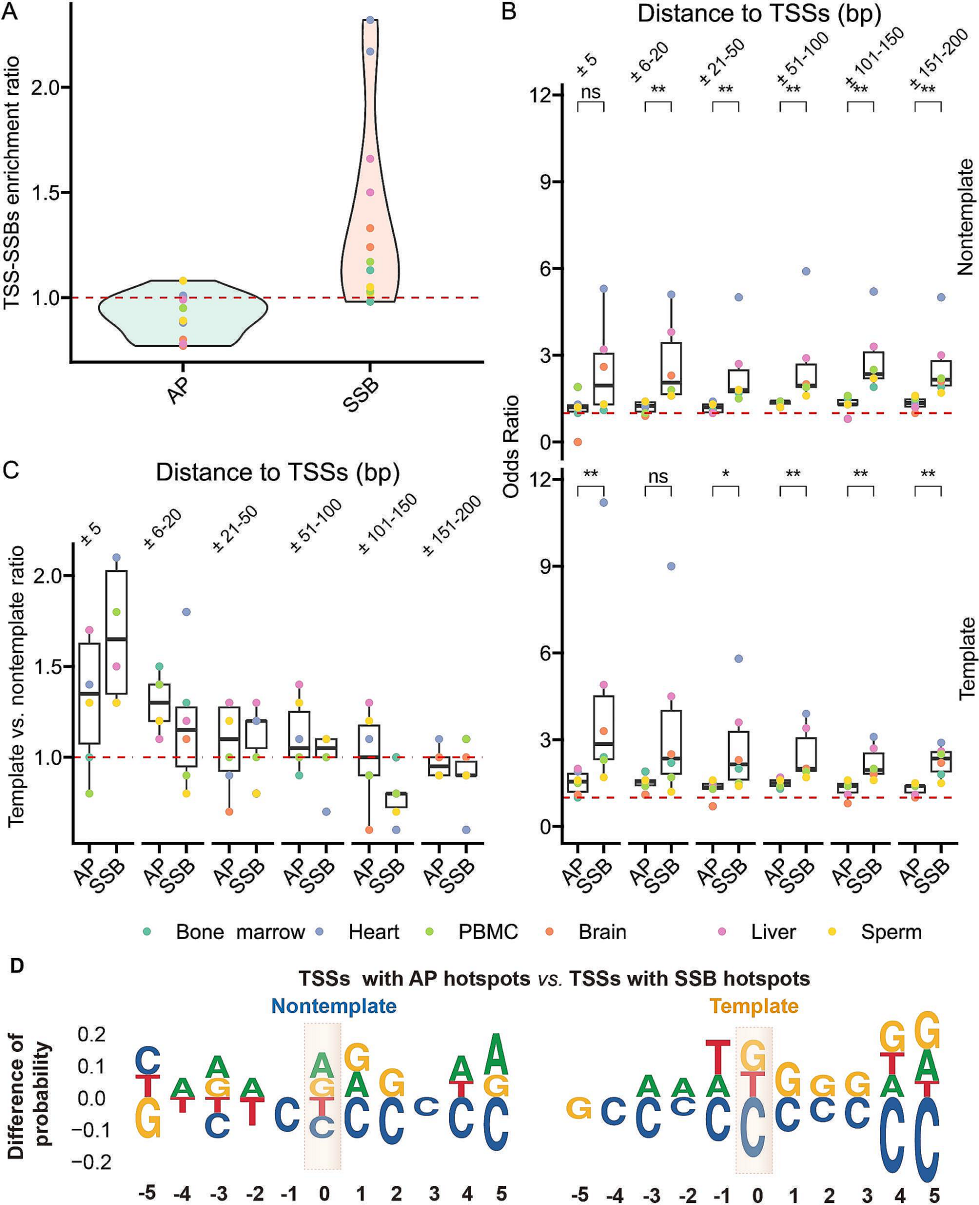
The genomic patterns of SSB hotspots near TSSs differ from those of AP site hotspots. (**A**) Violin plot distributions of the TSS-SSBs enrichment ratios (Y-axes) for the sample-shared SSB (pink) or AP site (green) hotspots (depth ≥ 1) in six tissues. Individual data points are shown. (**B**) Box plots of the odds ratios (Y-axes) of enrichment of sample-shared SSB or AP hotspots (depth ≥ 1) found on other template or non-template strands in the indicated (X-axis) non-overlapping distance bins to TSSs in the different tissues. (**C**) Template vs. nontemplate ratios (Y-axes) of sample-shared SSB or AP hotspots (depth ≥ 1) in each indicated (X-axis) distance bin and each tissue. (**B**, **C**) Green represents bone marrow, light blue represents heart, light green represents PBMC, orange represents brain, pink represents liver and yellow represents sperm. The asterisks above the horizontal lines denote the significance of the difference: *, ** represent respectively p-values < 0.05 and 0.01 (Wilcoxon Rank Sum Test), while “ns” mean no significant difference. Box plots indicate median (middle line), 25th, 75th percentile (box) and 1.5× interquartile range (whiskers) as well as individual data points. (**D**) The difference in the sequence compositions around TSSs that contain AP site hotpots within ± 5 bp vs. those that contain SSB hotspots within the same range on either template or non-template strand. Only bases with differences > 0.05 (5%, Y-axes) at each position are shown. Positive values on Y-axes indicate enrichment in the TSSs with the AP site hotspots. The TSSs are highlighted

Since, as mentioned above, AP sites have also been implicated in regulation of gene expression, we performed similar analyses for AP sites and their hotpots previously identified by our group [10] in same DNA samples that were used for generation of the SSB profiles. Interestingly, we found that the patterns for AP sites and their hotspots were markedly different from those found for SSBs. First, compared to SSBs, AP sites in general and AP site hotspots were significantly less enriched around TSSs in all distance bins and on either template or nontemplate strands (Fig. 7A and B, Supplementary Table 4, also see below). For example, the maximum TSS-SSBs enrichment ratio was 2.32 for the SSB hotspots, while only 1.08 for AP site hotspots (Fig. 7A, Supplementary Table 4). The maximum odds ratios of enrichment of the sample-shared hotspots on the template strand in the ± 151–200 bp and ± 5 bp regions around TSSs were 2.88 and 11.17 for SSBs and 1.50 and 1.98 for AP sites (Fig. 7B, Supplementary Table 4). The corresponding median values were 2.33 and 2.86 for SSBs and 1.38 and 1.51 for AP sites (Fig. 7B, Supplementary Table 4).

Second, AP site hotspots exhibited lower preference for the template strand of genes in the ± 5 bp windows around the TSSs. As shown on Fig. 7C, the median template vs. nontemplate ratio for such SSB hotspots reached 1.65 in the ± 5 bp bin around TSSs and gradually dropped to 0.92 in the ± 151–200 bp bin (Supplementary Table 4). However, the corresponding values for the AP sites were 1.30 and 0.95 (Fig. 7C, Supplementary Table 4).

Third, genes containing AP site hotspots in ± 200 bp windows around their TSSs exhibited significantly higher expression levels compared to the genes without them (Fig. 4, Supplementary Table 6). However, there was no increase in expression with the decrease in the distance between the AP site hotspots and TSSs that what observed for the SSB hotspots (Fig. 5, Supplementary Table 6, Methods). Fourth, the sequences in the ± 5 bp regions around TSSs of genes that contained AP site hotspots either on the template or nontemplate strands in these regions were enriched in purines (guanines and adenine), rather than cytosines, on the strands containing the AP sites (Fig. 7D, Supplemental Table 7). Specifically, the median fractions of guanines in the ± 5 bp windows were 34% and 32% for the TSSs containing AP site hotspots on the template or nontemplate strands respectively compared to 31% and 28% for the SSB hotspots (Supplemental Table 7). And the corresponding fractions of adenines were 22% and 21% for TSSs with AP site hotspots, which were also higher than 17% of adenine found on either strand in the case of the TSSs with the SSB hotspots.

In summary, in addition to biological implications (see Discussion), an important conclusion from these results is that while SSBs and AP sites were detected by SSiNGLe and SSiNGLe-AP techniques that share a large fraction of biochemical steps, their patterns of enrichment around TSSs were quite different. This result strongly argues that the enrichment properties of SSBs around the TSSs and the properties of genes that contain proximal TSS-SSBs reflect the true biology of the cell rather than a technical artifact (see Discussion).

### Base excision repair (BER) might contribute to the generation of TSS-SSBs

**Fig. 8:**
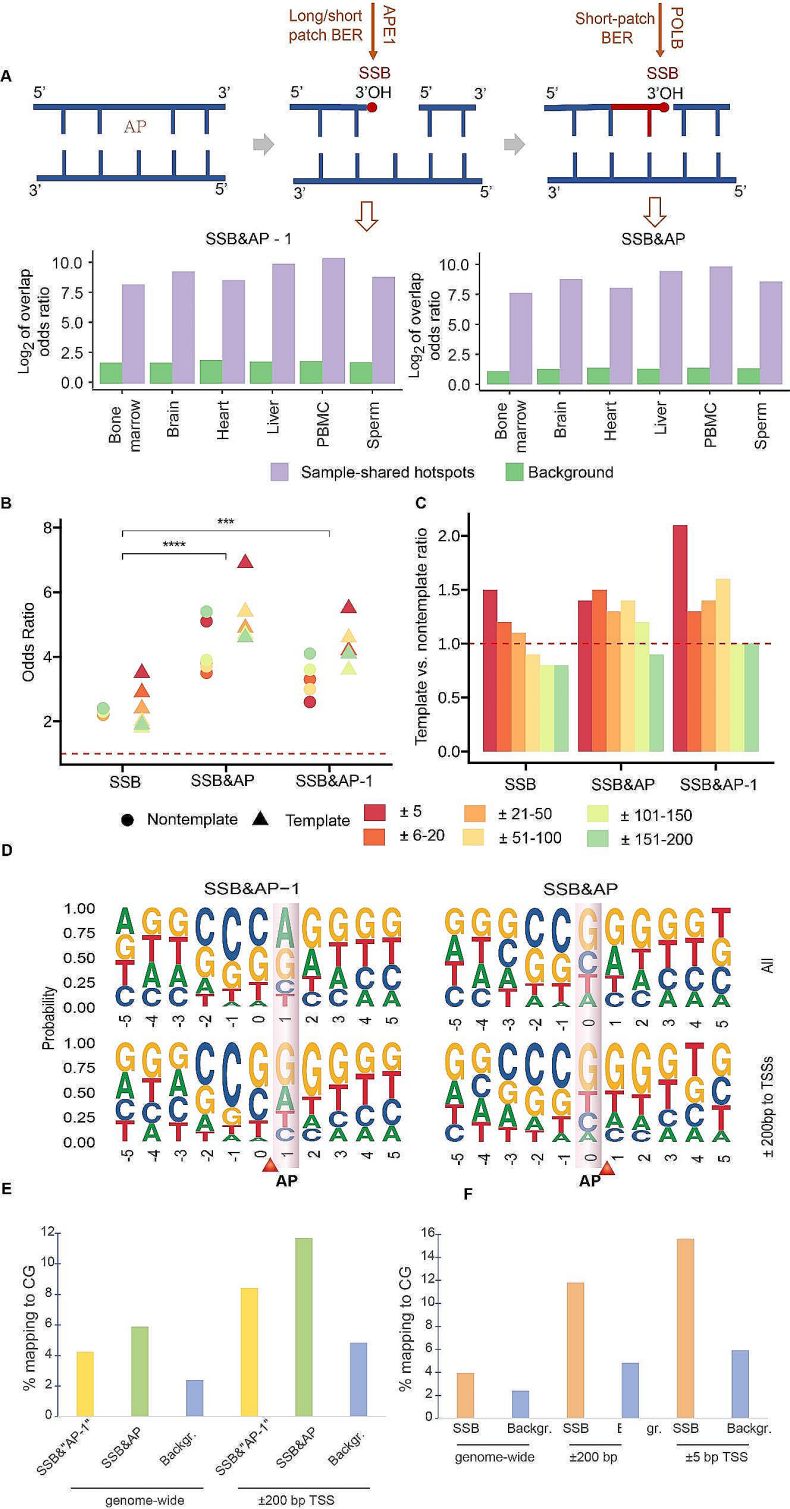
SSB hotspots overlap significantly with AP site hotspots. (**A**) Top, the diagram showing two types of SSBs with fixed genomic positions relative to those of AP sites that are generated during the repair of the AP sites by BER. Detecting the overlap between AP site and the SSB generated by APE1 requires shifting the genomic coordinate of the AP site upstream by 1 base and is therefore denoted as “AP − 1”. Bottom, the odds ratios of genome-wide overlap between sample-shared SSB hotspots and sample-shared AP hotspots (depth ≥ 1, purple), and the average odds ratios of overlap between all SSBs and AP sites (background, green) in each tissue. The odds ratios are shown on the Y-axes in the log_2_ scale. The left and right panels represent two different modes of overlap to detect the corresponding two types of SSBs shown in the top panel. (**B**, **C**) Odds ratios of enrichment (Y-axis, B) and the template vs. nontemplate ratios (Y-axis, C) of all SSB hotspots and those that overlap with AP site hotspots and found on other template or non-template strands in the indicated non-overlapping distance bins to TSSs. The red dashed horizontal lines represent odds ratios (**B**) or template vs. nontemplate ratio (**C**) of 1 that represent respectively no enrichment or no strand preference. The asterisks above the horizontal lines denote the significance of the difference: ***, **** represent respectively p-values < 0.001 and 0.0001 (Wilcoxon Rank Sum Test). (**D**) The sequence motifs around the sample-shared SSB hotspots that overlap with AP site hotspots genome-wide or within ± 200 bp to TSSs. The positions of the SSB hotspots are indicated by the red arrows and the corresponding overlapping AP site hotspots are highlighted. (**E**) Fractions of positions (Y-axis) of the SSB hotspots that overlap with AP site hotspots and map to CG dinucleotides genome-wide and within the ± 200 bp windows around TSSs. (**F**) Fractions of positions (Y-axis) of the SSB hotspots that map to CG dinucleotides genome-wide and within the ± 5 bp and ± 200 bp windows around TSSs. (**E**, **F**) “Backgr.” represents the background fractions of CG nucleotides in the corresponding mouse reference genomic sequence

Since one of the major pathways of generating SSBs is via BER-mediated repair of AP sites, it is conceivable that a certain fractions of SSBs and their hotspots, including those around TSSs, could be generated via this pathway. If so, it would be expected that AP sites that have not yet been repaired by BER would be found in the same genomic locations as the SSBs, but in different cells. This would be expected if these locations represented preferred sites of AP site formations, aka hotspots. To explore this possibility, we tested whether SSBs had tendency to overlap with AP site genome wide and around TSSs and whether this tendency was significantly higher for the hotpots. During BER, at least two SSBs are generated for each AP site with fixed locations relative to the site (Fig. 8A) [35]. One, SSB immediately upstream of the AP site produced by an endonuclease that recognizes an AP site, such as the mammalian APE1 protein (Fig. 8A). This SSB would be common to both short- and long-patch BER pathways [35]. Two, the downstream SSB that exists after the DNA polymerase fill-in step and prior to ligation [35]. In the short-patch BER, such SSB would be located immediately following the AP site (Fig. 8A), while in the long-patch BER, it’s position relative to the AP site would be variable [35]. Since our technique could be capturing both types of breaks, we performed overlap between the SSB and AP profiles in two different ways (Fig. 8A): (1) by shifting the coordinates of AP sites upstream by 1 base to detect the upstream breaks and, (2) by performing direct overlap to detect the downstream break generated by short-patch BER.

Interestingly, we detected statistically significant genome-wide overlap between SSBs and AP sites in each tissue and for both types of breaks (Fig. 8A, Supplementary Table 9). Moreover, the strength of the overlap increased significantly for the SSB hotspots compared to all SSBs in each tissue (Fig. 8A, Supplementary Table 9). However, the fraction of SSB hotspots that overlapped AP site hotspots was small: 0.11–1.51% across the six tissues genome-wide. Reciprocally, the fraction of AP site hotspots that overlapped SSB hotspots was also small and was in the range of 0.26–2.04% genome-wide. Still, since AP sites that contribute to SSB hotspots might be short-lived and therefore not detectable, and vice versa, the existence of significant overlap is indicative of BER being a contributing factor to the biogenesis of TSS-associated SSB hotspots, even though, it does not prove it (see Discussion).

We then tested whether SSBs that overlapped AP sites were more enriched in the proximity to TSSs. For this analysis, we merged all 71 samples and regenerated the hotspots for AP sites and SSBs shared by at least two samples, respectively, prior to the overlap since the number of overlapping hotspots around TSSs was too small in individual tissues. Just like for the hotspots generated for each tissue, the overlap between the SSB and AP site hotspots was highly significant in this analysis as well (Supplementary Table 9). Strikingly, the SSB hotspots that overlapped AP sites had much stronger enrichments around TSSs than all SSB hotspots and that enrichment increased in the immediate vicinity (± 5 bp) around TSSs (Fig. 8B, Supplementary Table 10). The SSBs that overlapped AP sites also had preference for the template strand in the ± 5 bp around TSSs (Fig. 8C, Supplementary Table 10). These patterns were true for overlaps with and without the + 1 shift (Fig. 8C, Supplementary Table 10).

Recently, it has been shown that active demethylation of methylcytosines via the BER pathway can be a major contributing pathway to generation of stable SSBs in mammalian cells [36, 37]. Therefore, we tested whether this mechanism could also be the major contributor to the SSB hotspots found in this work, especially those that overlap with AP sites and found near TSSs. Demethylation of a methylcytosine would create an AP site where cytosine is normally present in the reference genomic sequence and therefore, we tested whether the SSB hotpots that overlap AP site hotspots were enriched in cytosines. However, as shown in the Fig. 8D and Supplementary Table 11, such hotspots were enriched in purines (adenines or guanines) instead. Still, 5.89% or 4.24% of the SSB hotpots that overlapped AP site hotspots genome-wide either directly or with one base shift mapped to cytosines in the CG context (Fig. 8E). While representing a minority of hotspots, these fractions were higher than the expected 2.40% based on the fraction of CG dinucleotide in the non-repeat portion of the mouse genome (Fig. 8E). Furthermore, the corresponding fractions increased to 11.69% and 8.42 within the ± 200 bp windows around all TSSs compared to the expected 4.82% (Fig. 8E). Too few overlapping hotspots were found within the ± 5 bp windows for a meaningful analysis.

However, it is also conceivable that AP sites generated by methylcytosine demethylation are short-lived and thus do not represent AP site hotspots and would not be captured by the analysis above. In this case, since mammalian methylcytosines occur primarily in the CG dinucleotide context, we would have expected that SSB hotspots would be enriched in the CG context. Similar to the SSB hotspots that overlap the AP sites, all SSB hotspots do exhibit a preference for the CG context genome-wide: 3.96% (10,442 of 263,702) of SSB hotspots were found in either|CG or C|G context (‘|’ representing the location of the SSB), which was higher than the expected 2.40% based on the background found in the non-repeat portion of the mouse genome (Fig. 8F). Among the SSB hotspots found within the ± 200 bp and ± 5 bp windows around TSSs on either strand, the corresponding fractions of 11.8% (1,761 of 14,885) and 15.6% (150 of 960) were higher than the respective background fractions of 4.82% and 5.93%. Altogether, our results argue that BER-mediated repair of damaged purines appears to be a larger contributor to SSB hotspots than the active demethylation. Nonetheless, based on the higher than expected overlap with CG dinucleotides, the demethylation also appears to contribute to the pool of SSB hotspots found genome-wide and near TSSs (see Discussion).

## Discussion

We previously reported the existence of SSB hotspots in two human cell types, a malignant cell line and PBMC, and the enrichment of the hotspots around TSSs [11]. Here, by analyzing profiles of SSB breakome in 6 different normal mouse tissues, we have expanded on these initial observations to show that the existence of SSB hotspots and their enrichment around TSSs appears to be a common feature of normal mammalian cells. The enrichment of SSBs around mammalian TSSs, however, has not been detected with the only other high-resolution genome-wide SSB detection technique, GLOE-seq [34], which has been tested on a complex genome, such as human, and thus remains somewhat controversial. Overall, the following arguments support the notion that the observed enrichment of SSBs around TSSs represents a true biological phenomenon in the cell rather than a technical artifact. First, the profiles of SSBs used in this work have been obtained on purified genomic DNA [10]. This is an important consideration since other DNA break mapping studies have used crosslinked nuclei as the input material [6], which raises the issue of differential accessibility of cross-linked chromatin in different regions of genome to the enzymes that are used to tag DNA breaks. However, this issue does not exist when purified DNA is used since every break is equally accessible to the reagents used to detect it.

Second, interestingly, the TSS-related properties of SSB breakome were quite different from those of AP sites: (1) we found no enrichment of AP site hotpots in the ± 200 bp regions around TSSs relative to the ± 5,000 bp region background, (2) there was no progressive enrichment of AP site hotspots with the decreasing distance to TSSs, (3) the sequences in the ± 5 bp windows of TSSs containing AP site hotspots were enriched in guanines and adenines rather than cytosines and (4) there was no increase in gene expression as the distance between the AP site hotspots and TSSs decreased. Since AP sites were profiled using the SSiNGLe-AP technique that shares multiple steps with the SSiNGLe technique used to profile SSBs, if the enrichment of SSBs was a technical artifact, we would have expected to observe similar levels of enrichment in the AP sites as well, which was not the case.

Third, we observed significant correlations between two independent pairs of features of genes: (1) positive correlation between the presence of SSB hotspots and gene expression and (2) negative correlation between the distance between SSBs and TSSs and gene expression. Such correlations would not be expected if the enrichment of SSBs at TSSs was an artifact of the method. Furthermore, as mentioned above, the second correlation was not observed in the case of AP site hotspots.

Fourth, and perhaps one of the strongest arguments, is the big differences in the magnitude of SSB enrichment around TSSs among the different tissues. Since SSBs in all tissues were profiled using the same technique based on purified DNA, if the observed enrichment around TSSs was caused solely by an artifact of the procedure, we would expect to observe similar levels of enrichment in all tissues. However, this was not the case. Currently, the underlying biological reasons for the tissue-specific differences in the enrichment of SSBs and their hotspots around TSSs remain unknown. It is tempting to speculate that oxidative stress might be at least partially responsible in liver and heart that exhibit higher levels of enrichment. However, since the steady state levels of DNA damage are defined by the balance of the dynamics of DNA damage and repair, it is quite likely that other factors are involved, such as the expression of DNA repair factors, the accessibility of DNA to free radicals and DNA repair machinery in the context of local chromatin environment and others. This might also explain why brain, a tissue that also has a high oxidative burden, has a relatively low level of enrichment.

Presence of double-strand breaks (DSB) around TSSs has been documented in several studies [34, 38, 39]. While the SSiNGLe methodology can detect both SSBs and DSBs, the presence of the latter should be evidenced by DNA breaks present on both template and nontemplate strands. However, we observed a consistent preference for one of the strands, which is consistent with SSB-, rather than DBS-, derived signal. Overall, we believe that these arguments strongly support the *bona fide* nature of the enrichment of SSBs, and especially their hotspots, around TSSs.

Perhaps the most unexpected finding from this work was that the genes with SSB hotspots on the template strands in the immediate vicinity of TSSs had a tendency to have higher expression compared to the genes without them or genes with the hotspots further away from TSSs. A comprehensive in vitro study has shown that template strand SSBs in the vicinity of RNA polymerase III TSS can either affect the start site selection or drastically inhibit transcription depending on the distance from the TSS [31]. Furthermore, SSBs on the template strand were shown to block progression of RNA polymerase II [40] and prokaryotic RNA polymerases [41].

As mentioned above, transcription can generate DNA damage [12], therefore the TSS-associated SSBs and SSB hotspots could in fact represent by-products of transcription initiation. For example, the open chromatin environment could make the regions around TSSs of actively transcribed genes more accessible to DNA damage-causing endogenous factors, such as free radicals or cellular nucleases. Furthermore, the SSBs and their hotspots could be by-products of programmed DNA damage required for proper transcription initiation, such as BER-mediated demethylation of methylcytosines (see below) or topoisomerase activity [22]. If so, it would be logical to surmise that the abundance of such by-products would increase with increase in gene expression. Still, even if representing by-products of transcription, such SSBs would have biological relevance for at least two reasons. First, SSBs can be converted into DSBs [42], and thus can give rise to TSS-associated DSBs that have been shown to cause genomic rearrangements [38]. Second, SSBs can potentially lead to mutations [9], which would likely affect the regulation of gene expression when occurring in the proximity to TSSs.

On the other hand, the current study suggests that the relationship between SSBs and transcription initiation might be more complex than previously thought and raises a possibility that SSBs adjacent to TSSs, particularly those on the template strand, can stimulate transcription initiation. Interestingly, the stimulatory effect of SSBs on transcription initiation would then be similar to such an effect previously found for DSBs [43], and consistent with the growing albeit still controversial concept of persistent promoter-associated DNA breaks that have physiological relevance in regulation of gene expression [22, 44, 45]. Overall, additional studies are required to address the functional implications of the presence of SSB hotspots near TSSs.

The SSiNGLe method used in this work detects SSBs that have 3′-OH termini [9]. Such SSBs can be generated by several DNA repair processes that either convert multiple types of DNA lesions into 3′-OH SSBs or generate such breaks in the vicinity of DNA lesions [46]. The resulting SSBs are then typically repaired by dedicated DNA polymerases and ligases [46]. While these processes are normally highly efficient, it is conceivable that in certain genomic environments and under certain biological conditions, these processes could generate persistent 3′-OH SSBs at various stages of DNA repair, for example, immediately following the lesion recognition and endonuclease cleavage, during DNA polymerase extension or prior to the final DNA re-sealing stage by ligases. In fact, our results suggest that at least a fraction of SSB hotspots, including those near TSSs, might indeed be generated via BER from AP sites. Moreover, as hypothesized, we detected presence of two types of SSBs — those generated by the APE1 endonuclease, and the ones generated following the DNA polymerase extension prior to the ligation step.

A number of studies have reported that AP sites near TSSs can affect gene expression [23–25]. Such AP sites are generated via BER-mediated repair of oxidized guanines in the context G-quadruplexes [23–25]. Interestingly, the AP sites that overlap the SSB hotspots near TSSs are also enriched in guanines, consistent with the notion that the SSB hotspots are generated by BER-mediated repair of such AP sites. Interestingly, APE1-mediated cleavage of AP sites to generate SSBs was shown to be very slow in the G-quadruplex context [23–25]. In this respect, we found that TSSs containing AP site hotspots are enriched in guanines, potentially explaining why these AP sites persist and are slow to be repaired by BER. However, our data also suggests that demethylation of methylcytosines via BER could contribute a fraction of SSB hotspots. Still, while highly suggestive, these conclusions require wet lab confirmation based on profiling of AP sites and SSBs under the conditions where BER is inhibited to directly prove the relationship between hotspots of AP sites and SSBs found near TSSs and genome wide.

In addition to BER and other DNA repair pathways, 3′-OH SSBs could be generated via action of topoisomerases type II and IA [47], endogenous nucleases [48] and replication fork stalling [49]. While these breaks are typically either transient like in the case of topoisomerase-induced breaks or readily repaired by DNA damage repair pathways, a number of reports have shown that they can persist and regulate expression of nearby genes [22, 44, 45, 48]. Therefore, it is quite likely that some or all of these mechanisms contribute to the TSS-associated SSB hotspots described in this study.

## Conclusions

The enrichment of genes encoding proteins involved in DNA damage response and various RNA-related functions among the genes containing SSB hotpots in the immediate vicinity of TSSs brings a tantalizing possibility that SSBs at TSSs might serve as some sort of sensors of general DNA damage in the genome and thus upregulate these genes. In fact, changes in RNA processing have been shown to play critical role in DNA damage response [50] and thus would be consistent with this hypothesis. While AP sites have been implicated in control of gene expression, the stronger enrichment of SSB hotspots around TSSs compared to AP site hotspots suggests that the former type of lesion might play a stronger role in transcription control than the latter. Finally, the association of SSB hotspots with TSSs is consistent with the emerging theme of DNA damage as an epigenetic-like regulator of gene expression [25], and suggests that the true effects of various types of DNA damage on cellular physiology might be far more nuanced and complex that previously anticipated.

## Methods

### Genomic datasets

Coordinates of the AP sites and SSBs were obtained from our previous publication [10]. The coordinates of TSSs for all mouse annotated transcripts were extracted from the GENCODE VM23 database downloaded from the UCSC Genome Browser based on the mouse GRCm38/mm10 genome assembly. BEDTools (version 2.30.0) [51] was used to identify SSB and AP site hotspots and to calculate the overlaps between SSB/AP hotspots and various genomic windows around the TSSs.

### Generation and analysis of the simulated SSB data

For each sample, we randomly generated the same number of genomic SSB positions by the *sample* function in the R environment. Then the fractions of simulated SSBs shared between the samples in the same tissue or within the same sample (depth ≥ 2) were calculated. The simulations were performed in 100 iterations and in each round the hotspots were generated using the same procedure. The resulting fractions of the hotspots were used to make the plots in Fig. 1 and to calculate the significance of the occurrence of the hotspots.

### Aggregate plots, TSS-SSBs enrichment and odds ratios

Aggregate plot generation and calculation of the TSS-SSBs enrichment ratio for each tissue was identical to that used in our previous publication [11] and included the following steps:

1. The ± 5 kb region around each TSS was split into 500 non-overlapping 20 bp bins, and then the non-repeat fraction of each bin for each TSS was calculated. The average non-repeat fraction of each bin was then calculated based on the corresponding values of all bins for all TSSs.
2. For each AP/SSB site or hotspot, the distances to the TSSs were calculated and only the shortest distance was kept, and then used to assign the AP/SSB site or hotspot to one of the 500 non-overlapping 20-bp bins.
3. The normalized score representing the density of AP/SSB sites or hotspots in each 20-bp bin was identical to the *D*_*ij*_ metric defined in Cao et al. [11] and was used to make the aggregate plots.
4. The TSS-SSBs enrichment ratios of SSBs/AP sites or hotspots shown in the Figs. 2B and C and 7A were identical to the metric *R*_*i*_ defined in Cao et al. [11] and calculated for each sample *i* as follows:

$$ {R}_{i}= \frac{{M}_{i}^{200}/{M}_{i}^{5000}}{{L}_{i}^{200}/{L}_{i}^{5000}} $$

where M_*i*_^*200*^ and M_*i*_^*5000*^ are respectively the total numbers of unique positions of SSBs or hotspots within ± 200 bp or ± 5000 bp of TSSs in the sample; and L_*i*_^*200*^ and _*i*_^*5000*^ are respectively the total lengths of the non-repeated sequences within ± 200 bp and ± 5000 bp around the TSSs that do not include sequences that are adjacent to endogenous polyA stretches where SSBs and AP sites can not be mapped using SSiNGLe or SSiNGLe-AP.

(5) The enrichment of SSBs/AP sites or hotspots in various bins around TSSs shown in the Figs. 3B and C and 8B were calculated as odds ratios OR_*i*_ for each bin *i* as follows:

$$ {OR}_{i}= \frac{{M}_{i}/{T}_{i}}{{L}_{i}/LG}$$

where M_*i*_ is the number of positions of SSBs or hotspots mapping to the bin *i*; T_*i*_ is the total number of positions of SSBs or hotspots in a given sample; L_*i*_ is the total non-repeat length of the bin and LG is the total non-repeat length of the genome. L_*i*_ and LG do not include sequences that are adjacent to endogenous polyA stretches where SSBs and AP sites can not be mapped using SSiNGLe or SSiNGLe-AP.

(6) The overlap odds ratio (OOR_*i*_) between SSB and AP sites hotspots for each set *i* shown in Fig. 8A was similar to OR_*i*,_ and calculated as follows:

$$ {OOR}_{i}= \frac{{O}_{i}/{T}_{i}}{{M}_{i}/LG}$$

where *Oi* is the number of positions shared between hotspots of SSBs and AP sites; T_*i*_ and M_*i*_ are the total number of positions of hotspots of SSBs and AP sites, respectively and LG is the total non-repeat length of the genome. LG do not include sequences that are adjacent to endogenous polyA stretches where SSBs and AP sites can not be mapped using SSiNGLe or SSiNGLe-AP.

### Motif analysis

The motif analysis was based on the following steps:

1. Each base within the ± 5 bp region around each AP/SSB site or hotspot and on the same DNA strand was extracted using the “getfasta” function of BEDTools [51].
2. The fraction of each base (A, C, G, T) at each coordinate (within the ± 5 bp around target site) was calculated.
3. R package “Logoplot” was used to plot the fractions of each base at each position in the ± 5 bp region.

### RNA-seq analysis

The “rsem-calculate-expression” function of RSEM (version 1.2.28) was used to estimate the TPM value of each annotated transcript using a strand-specific approach [52]. Only one annotated transcript per gene was chosen at random to represent the expression level of that gene. The comparisons of the expression levels between genes that did and did not contain the sample-shared (depth ≥ 1) hotspots of SSBs or AP sites within 200 bp of their TSSs on either template or non-template strands presented in Fig. 4 were independently calculated for each tissue.

However, to estimate the relationship between the sample-shared (depth ≥ 1) hotspots of SSBs or AP sites located at various distances around TSSs shown in Fig. 5, the expression level of each gene was then calculated by averaging the TPM values of that gene across all samples of each tissue. This was done because the number of either SSB or AP site hotspots in each distance bin was relatively small and not sufficient to perform the analysis for each tissue. The genes were then classified hierarchically into 6 non-overlapping distance bins, ± 5 bp, ± 6–20 bp, ± 21–50 bp, ± 51–100 bp, ± 101–150 bp, and ± 151–200 bp based on the distances between SSB or AP site hotspots and the corresponding TSSs on either template or nontemplate strand. The genes containing hotspots on the same strand in two distance bins were assigned to the bin that was most proximal to the TSS. However, a gene that contained SSB or AP site hotspots on both template and non-template strands in the same distance bin was included in both template and nontemplate strand analysis. The comparison of gene expression levels among the 12 distance bins was based on the union of all genes in all tissues — genes that had an SSB or AP site hotspot within the same distance bin and on the same strand (template or nontemplate) in at least one tissue were grouped together. The p-values of the various comparisons were computed with the R package “ggpubr” (https://github.com/kassambara/ggpubr) and the figures were generated with the R package “ggplot2” [53].

### Electronic supplementary material

Below is the link to the electronic supplementary material.

Supplementary Material 1

## Data Availability

Data are provided within the manuscript or supplementary information files.
